# Psychological capabilities for salespeople’s sustainable work performance in financial services sector

**DOI:** 10.1057/s41264-023-00228-6

**Published:** 2023-04-17

**Authors:** Soo Yeong Ewe, Helen Hui Ping Ho

**Affiliations:** Department of Marketing, Monash University Malaysia, Jalan Lagoon Selation, Sunway City, 47500 Subang Jaya, Selangor Malaysia

**Keywords:** COVID-19, Financial services, Motivation, PsyCap, Psychological capability, Qualitative approach

## Abstract

The present study investigated the importance of psychological capabilities to support financial product salespeople in overcoming challenges and sustaining motivation and work performance during and after COVID-19 pandemic. Furthermore, this study suggests useful ways to develop the psychological capabilities. By using an interpretive phenomenological approach as the methodology, twenty financial product salespeople have been interviewed. The findings reveal that positive mindset, belief-in-oneself and not-giving-up are crucial psychological capabilities for salespeople’s sustainable work performance. These psychological capabilities match with the elements in the Psychological Capital Model. Positive mindset is associated with hope and optimism; belief-in-oneself is associated with self-efficacy, and not-giving-up is associated with resilience. The study also found approaches that help develop these psychological elements, including being proactive in seeking help and guidance, setting personal goal, and continuous learning. The findings contribute to the financial services and sales literature by providing a better understanding of how psychological capabilities help motivate financial product salespeople toward positive and sustainable work outcomes, and the ways to develop the psychological capabilities.

## Introduction

Salespeople’s sustainable work performance is crucial to sustaining economic performance of financial service sector. Most financial products (FP) are high involvement in nature and require a long period for customers to gain returns. Furthermore, financial products such as mutual fund and insurance are considered unsought products, which heavily rely on FP salespeople to personally approach potential customers and provide product recommendations that suit the customers to make sales (Claessens 2021). Due to the product nature, salespeople in the financial services sector experience inconsistent sales or frequent customers’ rejections that may affect their work motivation and performance. FP salespeople’s work performance could determine the sales of mutual fund and insurance, and consequently the sustainable economic performance of the financial services sector, and other related businesses and communities at large. Therefore, the sustainable work performance of FP salespeople is crucial to achieving sustainable economic development.

In the recent years, COVID-19 pandemic has disrupted the work performance of FP salespeople. They faced difficulty in finding new customers, and low demand for financial products due to an urgent need for liquidity among the existing or potential customers to survive the lockdown. These various challenges and constraints may negatively affect salespeople’s psychological capabilities and subsequently their work performance (Sharma et al. 2020). Studies into the psychological capabilities needed by salespeople and how to build them become paramount in advancing our understanding of psychological capabilities and sustainable work performance.

The existing literature on sustainable performance mainly focuses on performance at organizational (e.g., Fernando et al. 2019; Iqbal and Ahmad 2021) or sectoral level (e.g., Adams et al. 2014; Castellani and Sala 2010; Zaid et al. 2018), examined the impact of supply chain management (e.g., Hong et al. 2018), lean manufacturing practices (e.g., Kamble et al. 2020), innovation (e.g., Fernando et al. 2019), and human resource management (e.g., Zaid et al. 2018) on sustainable development. Among the limited studies on the contribution of leadership and positive psychology to sustainable organizational performance, organizational learning (Iqbal and Ahmad 2021), sustainable leadership (Iqbal et al. 2020a, b), psychological safety and psychological empowerment (Iqbal et al. 2020a, b) are found to be crucial in promoting sustainable performance.

The positive psychology literature documented psychological capital (PsyCap) (Luthans et al. 2007), which represents hope, efficacy, resilience, and optimism (known by the acronym H.E.R.O.), to be essential in improving employees’ wellbeing and ensuring sustainable work performance (Avey et al. 2010; Friend et al. 2016; Newman et al. 2014; Nolzen 2018). However, the study on salespeople’s PsyCap and sustainable performance is scarce (Friend et al. 2016). A recent study by Ewe and Ho (2022) suggest that PsyCap remains helpful to salespeople when coping with crisis such as the pandemic. However, the role of PsyCap in sustaining salespeople’s work performance in the financial services sector, and how PsyCap can be self-developed are not discussed in detail. Therefore, grounded theoretically in PsyCap, this study aims to fill this gap and explore other psychological capabilities for salespeople’s sustainable work performance in the financial services sector during and post COVID-19 pandemic. The study also aims to identify self-help strategies FP salespeople can use to enhance psychological capability for sustainable work performance. We asked two research questions. First, did psychological capabilities contribute to FP salespeople’s sustainable motivation and work performance during the pandemic? Second, how did the salespeople build such psychological capabilities to help themselves through difficulties?

Our contributions are two folds: We extend the PsyCap model by providing nuances on how psychological capabilities help FP salespeople overcome difficult situations and achieve sustainable motivation and work performance. We also identify self-help approaches that salespeople can undertake to build psychological capabilities, instead of relying on organizational support for the development of psychological capabilities. The theoretical contributions and practical implications of the study will benefit both academicians and practitioners.

The remainder of this article is organized as follows: The next section reviews literature of PsyCap and sustainable performance. This is followed by the research method and data analysis. The findings are then presented and their implications are discussed. The article ends with a concluding section that describes the study’s limitations and future research.

## Literature review

### Psychological capital (H.E.R.O) in the financial services sector

The theoretical framework that guides this study is Psychological Capital Model. The concept of psychological capital (PsyCap) developed by Luthans and colleagues (Luthans et al. 2006, 2007) represents an individual’s positive psychological state of development in terms of four component characteristics: hope, self-efficacy, resilience, and optimism (H.E.R.O.). Hopeful individuals are goal-directed, self-motivated, and they explore possible avenues to reach their pre-set goals (Friend et al. 2016). Someone with self-efficacy is confident and prevail over difficult tasks. Resilient individuals can recuperate from setbacks and failure promptly and effectively (Luthans et al. 2007). Optimistic people look upon future outcomes positively, and they will continue to broaden their efforts to achieve the positive outcomes (Lussier and Hartmann 2017). While PsyCap has some effects on work performance, employee wellbeing, and positive behavior in an organizational context, research on PsyCap in the financial services sales context has been limited (Friend et al. 2016).

The past studies of PsyCap in financial services sector include how HERO employee health management best practices scorecard influences organizational financial performance (Grossmeier et al. 2016), the effects of PsyCap on financial workers’ professional ethics (Zan 2020), the role of resilience on employee performance in banking industry (Cooper et al. 2019), and the effects of hope and optimism on organizational commitment among bank employees (Soni and Agarwal 2018). These past studies were conducted prior to the COVID-19 pandemic. It is unsure whether PsyCap or other psychological capabilities remain crucial to the performance of financial services employees during and post COVID-19 pandemic. Furthermore, the past studies focus on organizational intervention strategies such as best practice scorecard, human resource practices and social climate (e.g., leadership) in building financial services employees’ PsyCap, lacking research on the self-help approaches the employees can use in time of difficulties. Therefore, more research is needed to investigate how psychological capabilities motivate financial services salespeople to achieve sustainable work performance during and post COVID-19 period.

### Psychological capabilities and sustainable performance of salespeople

Sustainable work performance refers to high work performance that is persistent and sustainable. Employees with sustainable work performance demonstrate high level of energy, proactivity at work, and psychological well-being (Dorenbosch 2014). In this study, we define sustainable work performance of salespeople as long-lasting sales performance in which salespeople achieve sales targets, job satisfaction and customer satisfaction while maintaining energy, proactivity and well-being at work, and the work performance promotes sustainable economic development of the organization and financial services sector. Sustainable work performance can be achieved when salespeople are equipped with adequate job resources (such as autonomy, performance feedback, task significance, and social support), personal resources (such as PsyCap), and recovery opportunities to meet job demands (de Jonge et al. 2014; de Jonge and Peeters 2019; Iqbal et al. 2021). The strength of individual salesperson psychological capabilities (i.e., personal resources) contributes to the sustainable work performance of salespeople (Nolzen 2018). Positive psychological capabilities contain positive agentic capacity that is critical to human motivation and performance in organizations (Avey et al. 2010).

In the existing literature, salespeople’s sustainable work performance can be affected and determined by employers, supervisors and salespeople themselves. Employer’s strategies to influence salespeople’s psychological capability for better work performance include mindful relationship management (Zafari et al. 2020) and effective event communication (Luu 2021). Support from supervisors such as creating an ethical climate (Wimbush and Shepard 1994), giving autonomy and feedback (Bakker and Demerouti 2007), sustainable leadership (Iqbal and Ahmad 2021) and knowledge sharing (Matsuo and Aihara 2022) can result in more ethical sales behaviors and higher work capabilities. In terms of salespeople’s psychological resources, wisdom (Oh and Oh 2021), grit (Dugan et al. 2022), emotional intelligence (Bande et al. 2015), psychological safety and psychological empowerment (Iqbal et al. 2020a, b), patience, consideration and responsibility (Ewe and Ho 2022), and perseverance (Belschak et al. 2006) are found to improve salespeople’s work performance. These various organizational strategies and salespeople’ psychological resources contribute to salespeople’s sustainable work performance. Nevertheless, there are limited studies examining salespeople’s self-help efforts in building psychological capabilities to achieve sustainable work performance during the COVID-19 pandemic, especially in the context of financial services sector. Therefore, our study addresses this gap by examining the role of psychological capabilities in sustaining FP salespeople’s work performance during and after the pandemic, and how FP salespeople build these capabilities themselves.

## Research method

We adopted interpretive phenomenology to understand the lived experiences of FP salespeople during the first COVID-19 lockdown in Malaysia, particularly the lockdown’s impact on them and the approaches they took to overcome challenges and sustain work performance. Phenomenology, in general, is both a philosophy and methodology that seeks to understand experiential phenomena, that is, the “essence” of the experiences of a conscious person who has undergone them (Giorgi 2009). It is an approach that closely involves the research participants and that focuses on the precise analysis of how a participant experienced a phenomenon. Using interpretive phenomenology, we examined, interpreted and co-created meanings (Smith and Eatough 2007) with the FP salespeople of their experiences dealing with disruptions posed by the COVID-19 pandemic, and how they had sustained work motivation and performance during this challenging time.

### Data collection and participants

We used purposive sampling and snowball sampling to find and recruit participants for our study, after we had obtained the research ethic clearance from the University. The participants were identified and chosen through referrals from our contacts. In addition, informants who had been interviewed were asked to recommend their contacts who fit the selection criteria. In both sampling methods, we contacted the informants only after they had expressed their interest in taking part in the study. We interviewed a total of 20 participants. Nine are unit trust consultants and 11 are insurance agents who mainly sell life and medical insurance. The participants are a mixed group of experienced and inexperienced salespeople. Table 1 illustrates the range of participants by their demographic information (9 females, 11 males), their positions in the respective agencies (12 agency supervisors/managers, 8 regular agents), and the number of years they had been selling unit trusts or insurance (3–30 years). Each participant was given a pseudonym to maintain their confidentiality.

**Table 1:** Participants’ profile

No.	Pseudonym	Position	Sector	Sex	Age (2020)	Sales experience (in years)
1	Albert	A	IN	M	24	4
2	Belle	A	IN	F	38	14
3	Chloe	A	IN	F	35	5
4	Dustin	A	IN	M	33	7
5	Emily	AM	UT	F	47	15
6	Gary	GM	IN	M	39	17
7	Hannah	A	IN	F	27	3
8	Jerome	A	IN	M	32	3
9	Kevin	A	IN	M	31	7
10	Lillian	GAM	UT	F	53	30
11	Maya	AS	UT	F	48	4
12	Neelesh	AM	UT	M	48	12
13	Rohan	GAM	UT	M	43	10
14	Scott	GM	IN	M	40	18
15	Susan	A	IN	F	38	6
16	Terrence	UM	IN	M	24	4
17	Wendy	AS	UT	F	45	13
18	Wilson	AS	UT	M	42	7
19	Winnie	GAM	UT	F	53	6
20	Yuvan	AM	UT	M	56	14

*A* Agent, *AM* Agency Manager, *GM* Group Manager, *GAM* Group Agency Manager, *AS* Agency Supervisor, *UM* Unit Manager, *IN* Insurance, *UT* Unit trust, *M* Male, *F* Female, *S* Single, *M* Married

We conducted virtual, semi-structured in-depth interviews through Zoom during the first lockdown period in Malaysia. Both researchers conducted the interviews using an interview guide to ensure that key questions were asked across the multiple participants (Taylor et al. 2015). We started the interviews with open-ended questions (Lauterbach 2018), such as “How long have you joined the financial services sector?” “How did you join the sector?” The intention of asking these open-ended questions was to create a relax interview environment in which the participants could be more opened subsequently to share their experiences of lockdown, the impacts of lockdown on their sales performance, and their conscious efforts overcoming the sales challenges. Some subsequent questions included “What challenges did you face during the lockdown period?” “How did you carry out your work during the lockdown?” “How did you overcome the difficulties or challenges in selling the financial products during this period?” Following the interpretive phenomenological interview approach (Dahlberg and Dahlberg 2020), we listened attentively to the participants’ narratives, probed into their psychological functioning and asked follow-up questions after the participants had expressed the use of positive psychology to overcome challenges. Some probing and follow-up questions were “You mentioned that positive thinking is important. Can you explain it further?”, “What makes you move forward?”, “What are the values that help you achieve success in the future?” The interviews ranged from 35 min to 2 h 30 min. We collected a total of 21 h and 16 min of interview data.

### Data analysis

By using the interpretive phenomenological analytic (IPA) approach, we focused on and made sense of the participants’ lived experiences during the COVID-19 pandemic. When analyzing the interview data, we engaged in an ongoing “back and forth movement, of questioning and re-examining of the text, results in an ever-expanding circle of ideas about what is might mean to be” (McConnell-Henry et al. 2009, p. 11). This IPA approach helped us unfold and constitute meanings from the participants’ experiences, and brought forth our understanding of their lifeworld (Suddick et al. 2020). From the data, we identified the PsyCap elements that had influenced the participants’ work and life, and the approaches used by the participants to build the psychological elements. Table 2 lists the steps we undertook in the data analysis process. Table 3 demonstrates the development of themes from descriptive codes and some examples of quotes by the participants.

**Table 2:** Interpretive phenomenological analysis (IPA) process

Process of analysis	Level of analysis	Description of analysis
Familiarization/gaining insight	Reading the transcript	Read a single transcript a few times to get an overall perspective of the narrative given by the participant
Immersion and sense-making	Diagnosing the case	Code the transcript line by line, paying close attention to the participant’s experiential claims and comments
Categorization	Developing intra-case categories and themes	Consolidate the codes to form emerging categories. Cluster categories together, refine, and condense them to form themes for the case. Repeat the same procedure for all interview transcripts
Association/pattern recognition	Developing inter-case themes and master themes	Look for patterns across all structure of themes. Identify similarities and differences. Develop and finalize master themes that cover all cases
Interpretation/representation	Writing up	Transform shared narratives into writing while allow unique cases to re-emerge. Use participants’ excerpts from the interview as evidence
Explanation and abstraction	Enfolding literature	Incorporate theories into discussion of participants’ narratives. Engage in iterative and comparative process between theories and data

Adapted from Cope (2011)

**Table 3:** Development of themes from descriptive codes

Themes	Descriptive codes	Examples of interview excerpts
Psychological capabilities	Positive mindset	*I try to think it in a positive way. Yeah, because once you think negatively, everything also cannot (Scott)*
		*During pandemic, the main thing is not to worry, don’t look at too many negative things. The most important thing is positive energy. See the good from the bad (Yuvan)*
		*I believe that there are two sides to everything. When something bad happens, there must be something good too (Hannah)*
	Belief-in-oneself	*You don’t care how long you can get the return, you stick to your plan, you believe you can do it, you believe you can achieve the goal then you can do it (Wilson)*
		*I always believe… I want to groom more leaders to become agency manager (Winnie)*
	Not-giving-up	*Believing that everything has a solution, as long as we put in effort. We do not have to give up (Susan)*
		*I have decided to continue as I now have a positive mindset … this had prevented me from giving up (Terrence)*
Approaches to building psychological capabilities	Being proactive in seeking help and guidance	*I was a bit lost, I didn’t know what to do. Then, I consulted some seniors for advice. I even watched some of the sharing sessions from the more successful seniors (Hannah)*
		*He is someone that I will seek advices from even though he is not my manager (Kevin)*
	Setting personal goals	*There is a target that I want to achieve. It is an achievement that I want to do; it’s my goal. (Hannah)*
		*We must know what exactly that we really wanted (Kevin)*
		*As for next year, I planned to get myself an award from my company (Albert)*
	Continuous learning	*At my age now, I’m still learning every day, I thank God for giving me the chance to learn, to let me continue growing. If it weren’t for the 3 months of the MCO, I wouldn’t be able to talk to you on Zoom now (Lillian)*
		*We have to learn how to do online transactions. For some elder consultants, they said they don’t know, I told them that they need to learn. Nobody is born with knowing how to do things. We have to learn until we are old, lifelong learning (Yuvan)*

## Findings and discussion

The COVID-19 pandemic and the stringent controls of social activities had negatively influenced the work performance of the participants and their psychological well-being. The challenges and psychological impact can be summarized as follows: The participants felt that carrying out their role as FP salespeople during the pandemic became more difficult, as most businesses were closed and people had to work from home. Most of them found it hard to visit shops and potential customers during the pandemic. The participants were also anxious because the customers were less willing to invest during the pandemic. Some participants lost direction in their work and were unsure whether they could keep their job. The mental stress due to these challenges was tremendous, as it had affected the participants’ sleep, daily routines, and motivation to work.

### Psychological capabilities for sustainable work performance

The participants highlighted several elements of psychological capabilities that had helped them overcome the negative psychological impacts on work performance and the grim outlook in view of the pandemic. The psychological capabilities include positive mindset, belief-in-oneself, and not-giving-up. We found these elements match with the components of PsyCap (i.e., H.E.R.O) despite they were named differently by the participants. We also found that participants with more experiences in the financial services sector possess a higher level of psychological capabilities that help sustain their work performance, while the influence of other demographic factors such as age and gender on the participants’ level of psychological capabilities is not evident. We present the three psychological capabilities and the supporting quotes from the participants below.

#### Positive mindset

Most of the participants agreed that having a positive mindset is critical in the sales profession. Positive mindset produces positivity that subsequently leads to positive work behaviors among salespeople. In general, positivity is “a kind of attitude that includes thoughts, words, and images that help growth, development, and success” (Bakioğlu et al. 2020, p. 2). Salespeople with a positive mindset see positivity in dire situations. They are hopeful and optimistic of the future and are willing to try different approaches to achieve goals. Terrence said that having a positive mindset had given him hope so he did not give up easily when he was faced with sales challenges during the pandemic. Susan also perceived that “everything has a solution” with a positive mindset. She was willing to exert more effort to achieve sales target despite difficulty.

In contrast, negative mindset causes pessimism and can be detrimental to salespeople’s motivation. Salespeople with negative mindset may feel that “everything also cannot [be done]” (Scott) because they are “scared” and “worry about not having sales” (Yuvan). The negative mindset can hamper salespeople from seeing opportunities. Therefore, salespeople need to have positive energy (Yuvan) and optimism (Kevin) in the face of difficulties. They also need to stay away from those negatives others who only see pandemic as a threat to their sales performance (Lillian). To think positively and to identify opportunities from the dismay situations, salespeople have to adjust their mindset, as Susan explained: “I knew that I needed to change my mindset, because it’s completely different… I spent some time doing that.”

Having a positive mindset and adjusting one’s mindset to see only positive developments or opportunities can be a strong psychological capability that moves salespeople forward and produce sustainable work performance during difficult times. From the participants’ sharing, we can deduce that positive mindset encompasses the element of hope and optimism as found in the past PsyCap studies (Friend et al. 2016; Luthans and Youssef-Morgan 2017). With a positive mindset, salespeople can become hopeful, optimistic, and confident in solving problems (McLean and Dixit 2018) rather than being easily defeated by negative life or work circumstances. Having a positive mindset also enables salespeople to engage in innovative work behavior (Pukkeeree et al. 2020) or seek help from others when necessary. The finding adds on to the limited sales and financial services literature examining the constructs of hope and optimism. The finding also reaffirms the importance of these psychological capabilities for salespeople to achieve sustainable work performance.

#### Belief-in-oneself

In addition to positive mindset, belief-in-oneself emerged as another element of psychological capability that FP salespeople require when they face changes and new challenges. Individuals with a strong belief-in-oneself possess the confidence in their capability to act and achieve desired goals and success. Wilson said: “When you believe you can do it, you believe you can achieve the goal, then you can do it.” Scott gave a similar account: “Believe [in] yourself [that] you can do it, believe that insurance can help people.”

Possessing a strong belief-in-one’s capability to achieve sustainable sales performance goes beyond giving oneself a pep talk. Salespeople must acquire essential product knowledge and critical skills (such as communication and selling skills) to perform their job with confidence. Emily highlighted, “You need to have that technical knowledge in the unit trust, only then you know how to advise your clients what to do.” Lillian also concurred with Emily: “You must have the knowledge base after you are trained.” The acquisition of the right financial and technical knowledge requires salespeople to attend trainings provided by the companies, and “to do homework” by “study[ing] the market… all the knowledge about the funds” (Rohan). In addition, the participants ought to learn virtual selling and technological skills to enhance their confidence and that of their team to continue selling during the COVID-19 pandemic (Belle, Terrence).

From our analysis, we reckon that the belief-in-oneself element resonates with self-efficacy of the PsyCap model (Alessandri et al. 2018). In addition, our study found a relationship between belief-in-oneself and learning, i.e., that participants’ self-efficacy can be enhanced through continuous learning of new knowledge and skills. While Lee and Welliver (2018) found the relationship between continuous learning opportunities and salesperson performance to be non-significant, this study suggests that continuous learning may increase job performance through an increase in self-efficacy.

#### Not-giving-up

Salespeople should not give up easily in the face of rejection if they wish to succeed in the field. Our participants often face rejections because some prospects may perceive them as serving their own interests (Maya), or they would not serve the prospects over the long term (Kevin). Some prospects also view insurance as a “fraud which intends to cheat their money” (Kevin). These misconceptions about FP salespeople’s lacking integrity and honesty were exacerbated during the COVID-19 pandemic when most people had become more cautious in their spending. Scott shared his experience of rejections: “I faced a lot of [rejections]… There were a lot of mental challenges, and it was very easy to feel [down], because people usually reject first, and they need some time before they start to accept us. That period of time was a tough time to pass.”

Nevertheless, the participants refused to give up and found different ways to rebound from rejections. This not-giving-up psychological capability is aligned with the resilience component in the PsyCap literature (Nolzen 2018). We also found that not-giving-up can be extrinsically driven. FP salespeople can be motivated by their customers who have chosen to trust them, especially when the relationship was initiated when they were strangers to each other, and have developed a trusted relationship over time. Giving up will “disappoint” (Kevin) and “let down” (Scott) the customers who have trusted and supported them. Linhart and Stotz (2021) established that salespeople’s integrity and customer orientation play a main role in building interpersonal trust. We surmise that the trust could further enhance salespeople’s ability to rebound when facing challenges.

The three psychological capabilities (positive mindset, belief-in-oneself, and not-giving-up) found in our study, albeit were named differently by the participants are in line with the H.E.R.O. components in the past studies of PsyCap (Alessandri et al. 2018; Avey et al. 2010; Luthans et al. 2007; Nguyen and Nguyen 2012). Participants with a high level of PsyCap were found to be positive toward future development and were confident of their capability to meet challenges. They also did not give up and made efforts to sustain and enhance their work performance.

### Approaches to building psychological capabilities

Given the importance of psychological capabilities to salespeople’s sustainable motivation and work performance, we identified several self-help approaches initiated by the participants to build psychological capabilities. The approaches include being proactive in seeking help and guidance, setting personal goal, and continuous learning.

#### Being proactive in seeking help and guidance

To develop positive mindset, belief-in-oneself and not-giving-up attitude, our participants sought help and guidance proactively from their group/unit leaders and/or seniors (i.e., experienced salespeople). This self-help approach through communicating with the leaders and seniors enables inexperienced salespeople to receive communal support (Woodstock 2007), and to access the former’s well-established and useful knowledge, resources and network. The leaders and/or seniors can be an inspiration or a role model to new and inexperienced salespeople. The advice, encouragement and moral support from leaders and/or seniors can help motivate salespeople, enable them to develop the right mindset, confidence, and selling skills, as well as to formulate selling strategies (Albert, Chloe, Kevin, Terrence). The following excerpt provided by Terrence demonstrates the role of senior’s guidance on his motivation to continue work: “During the first two weeks of [lockdown], I became lazy… I talked to a senior of mine, whom I admire a lot, and he shared with me some ideas on how to start work in this new normal…”.

After talking to their leaders and/or seniors and listening to the advice and sharing, the participants felt “energized” (Wilson), “enlightened” (Hannah), and were determined to persevere in the face of rejections and sales challenges, as evidenced in Wilson’s quote: “[They] transferred some positive mindset or sharing to make us get back the energy to go further, to do sales.” From the participant narratives, we can infer that leaders and/or seniors in the same organization can serve as a community of practice that helps educate and guide salespeople for sustainable work performance (Matsuo and Aihara 2022).

#### Setting personal goal

The second self-help approach to develop a not-giving-up attitude is to set specific personal goals. Personal goals are personal strivings (Seaton and Beaumont 2015). They are the accomplishments individuals wish to attain, and vary among individuals. Setting personal goals instead of abiding by organizational goals or following others’ goals motivates salespeople. Kevin said, “Most of them are unclear about their goals, they just follow how others do it… It is important for the individuals to know what they are looking for.” For some participants, their personal goal is to achieve a certain income level or lifestyle. Therefore, setting a goal to attain a desired sales figure or a certain number of customers within a specific timeframe motivates them to persevere through adversity and achieve sustainable work performance (Jerome, Maya, Winnie,). Wilson shared that he overcame difficulties by focusing on his goal: “My goal… I want to get this kind of income, I want to get this kind of lifestyle, then I have to stick to the plan.” Albert also reflected that his goals of getting awards and recognition from the company propelled him to work hard and stay resilient as he declared, “I wanted to prove that I am able to achieve the things I aimed for.” Setting personal goals thus increases FP salespeople’s motivation, resilience and goal attainment which subsequently contribute to sustainable work performance.

#### Continuous learning

The third self-help approach is continuous learning. This approach is particularly pertinent to the development of belief-in-oneself. As highlighted in our discussion of psychological capabilities, acquiring new knowledge and skills has a positive effect on self-efficacy, which in turn can sustain or increase salespeople’s job performance. Salespeople who continue learning in the job are learning-oriented. They are motivated by sales challenges and are willing to adapt their selling strategies and behaviors to suit the customers or selling situations (Park and Holloway 2003; Sujan et al. 1994). Continuous learning is critical to FP salespeople’s development, particularly when they must sell a variety of financial products and deal with customers from different backgrounds with different needs.

Our participants engaged in learning continuously. They learned about the new funds, the new insurance schemes, the emerging financial markets, and the use of technology (such as social media, virtual conference software) for virtual selling and online transactions. The participants shared that they had actively attended trainings and seminars organized by their companies during the pandemic to sustain their motivation and continue selling (Chloe, Dustin, Hannah, Wilson). In addition, they had searched for online resources, including free software and financial market analysis tools, to support their sales efforts (Lillian, Yuvan), instead of solely depending on sales materials provided by the companies. These self-initiated learnings by the participants build their confidence to continue selling despite the pandemic. Lillian, a successful group agency manager with 30 years of sales experience, emphasized the significance of continuous learning for salespeople’s sustainable work performance: “There is no shortcut in the process of learning. You cannot skip classes, you must learn…Your learning attitude is the most important thing… You cannot stop learning. When you face problems, you have to find a way to overcome it.”

## General discussion

This study aims to explore whether psychological capabilities contribute to FP salespeople’s sustainable work performance during the pandemic, and how salespeople can build them. We found that positive mindset, belief-in-oneself and not-giving-up are essential psychological capabilities to sustain work motivation and performance during the pandemic. Furthermore, the three psychological elements are found to correspond to the H.E.R.O components in the PsyCap model: positive mindset links to hope and optimism, belief-in-oneself links to self-efficacy, and not-giving-up links to resilience. While Luthans and Youssef-Morgan (2017) emphasized the combination of H.E.R.O. in producing positive outcomes, our study provides further nuanced insights to the existing PsyCap model by delineating and explicating how the three corresponding psychological capabilities help FP salespeople overcome challenges during the pandemic and achieve sustainable work performance in the financial services sector.

Besides, we discovered the self-help approaches that salespeople can use to develop such important psychological capabilities. The approaches include being proactive in seeking help and guidance, setting personal goal, and continuous learning. The three approaches can be applied at the individual level without the employers’ intervention. These findings of self-development strategies add on to the past studies that mainly focused on intervention strategies at the organizational level (e.g., Hartmann and Lussier 2020; Luthans and Youssef-Morgan 2017; Luu 2021).

## Theoretical and practical implications

The present study contributes to the financial services and psychological capital literature by providing useful insights into the emerging psychological capabilities, and the multiple individualized approaches that FP salespeople can use to develop such capabilities. Our findings evince that psychological capabilities drive salespeople in financial services sector to overcome challenges during the COVID-19 pandemic for sustainable work performance. This study extends the past findings on positive psychology (Luthans et al. 2006) and sustainable performance (Iqbal et al. 2020a, b; Iqbal and Ahmad 2021; Iqbal et al. 2020a, b) by highlighting the importance of psychological capabilities in sustaining salespeople’s motivation and work performance in the financial services sector. The study also extends earlier research of positive psychological interventions (Dello Russo and Stoykova 2015; Luthans et al. 2006; Luthans and Youssef-Morgan 2017) by identifying several self-help strategies. Future researchers in the same research area can extend our study through validating the relationships between the self-help approaches and the corresponding psychological capabilities using a quantitative approach. In addition, they can consider the factors that may limit the effects of those approaches on building the psychological capabilities.

The present study also supplies insightful practical implications. Salespeople in financial services sector should be aware of the importance of psychological elements in sustaining their motivation and work performance. They are advised to engage the proposed self-help approaches to enhance their psychological capabilities. For instance, salespeople should cultivate a positive mindset by reading positive quotes, writing good words, noting good own experience every night before going to bed, speaking positive words, and reminding themselves to think positively. Besides that, salespeople should approach their leaders and seniors in the same field for advice and assistance whenever they face difficulties such as rejections by customers or prospects. Guidance from leaders and seniors may help solve their problems or reduce negative thoughts sooner than if they overcome the obstacles themselves. Setting specific personal goals (e.g., achievement, or happiness) may motivate salespeople to move forward and not to give up easily in achieving success despite difficulties. Salespeople should set and revise when necessary their short- and long-term goals for their career regularly. Furthermore, salespeople should believe in themselves and value continuous learning to enhance their self-efficacy in work. They can sign up for courses and seminars that can enhance their knowledge and skills in the financial services context.

## Limitations and future research

This study has its limitations. First, the scope of the study is within salespeople in the financial services sector. Therefore, the findings may be more applicable to the financial services context. Second, this article focuses on the discussion of psychological capabilities and how they contribute to sustainable work performance for salespeople in the financial services sector. It is possible that other psychological elements may also influence their performance. Therefore, future research may explore if other psychological elements can motivate the salespeople in the financial services sector to sustain good performance. Furthermore, our study examines self-development approaches in building psychological capabilities. Future research may enquire into the social context in building psychological capabilities, for example, the adoption of community of practice. Additional research may also investigate the moderating effects of demographic variables on the relationship between psychological capabilities and sustainable work performance.
